# An interdisciplinary mixed-methods approach to developing antimicrobial stewardship interventions: Protocol for the Preserving Antibiotics through Safe Stewardship (PASS) Research Programme

**DOI:** 10.12688/wellcomeopenres.15554.1

**Published:** 2020-01-14

**Authors:** Laura Shallcross, Fabiana Lorencatto, Christopher Fuller, Carolyn Tarrant, Jonathan West, Rosanna Traina, Catherine Smith, Gillian Forbes, Elise Crayton, Patrick Rockenschaub, Peter Dutey-Magni, Emma Richardson, Ellen Fragaszy, Susan Michie, Andrew Hayward, PASS Research Group

**Affiliations:** 1 University College London, London, UK; 2 University of Leicester, Leicester, UK; 3 Royal College of Art, London, UK

**Keywords:** Antimicrobial-stewardship, behaviour-change, interdisciplinary, ethnography, epidemiology, user-centred design

## Abstract

Behaviour change is key to combating antimicrobial resistance. Antimicrobial stewardship (AMS) programmes promote and monitor judicious antibiotic use, but there is little consideration of behavioural and social influences when designing interventions.  We outline a programme of research which aims to co-design AMS interventions across healthcare settings, by integrating data-science, evidence- synthesis, behavioural-science and user-centred design.

The project includes three work-packages (WP):

**WP1 (**Identifying patterns of prescribing):  analysis of electronic health-records to identify prescribing patterns in care-homes, primary-care, and secondary-care. An online survey will investigate consulting/antibiotic-seeking behaviours in members of the public.

**WP2** (Barriers and enablers to prescribing in practice): Semi-structured interviews and observations of practice to identify barriers/enablers to prescribing, influences on antibiotic-seeking behaviour and the social/contextual factors underpinning prescribing. Systematic reviews of AMS interventions to identify the components of existing interventions associated with effectiveness. Design workshops to identify constraints influencing the form of the intervention. Interviews conducted with healthcare-professionals in community pharmacies, care-homes, primary-, and secondary-care and with members of the public. Topic guides and analysis based on the Theoretical Domains Framework.  Observations conducted in care-homes, primary and secondary-care with analysis drawing on grounded theory.  Systematic reviews of interventions in each setting will be conducted, and interventions described using the Behaviour Change Technique taxonomy v1. Design workshops in care-homes, primary-, and secondary care.

**WP3** (Co-production of interventions and dissemination). Findings will be integrated to identify opportunities for interventions, and assess whether existing interventions target influences on antibiotic use. Stakeholder panels will be assembled to co-design and refine interventions in each setting, applying the Affordability, Practicability, Effectiveness, Acceptability, Side-effects and Equity (APEASE) criteria to prioritise candidate interventions.

Outputs will inform development of new AMS interventions and/or optimisation of existing interventions.  We will also develop web-resources for stakeholders providing analyses of antibiotic prescribing patterns, prescribing behaviours, and evidence reviews.

## Introduction

Since the 1940s, antibiotics have transformed our ability to treat bacterial infections. However, antibiotic overuse has allowed the evolution of antibiotic resistant bacteria that can survive the effect of an antibiotic. The emergence of antimicrobial resistance (AMR) is a global problem which is estimated to cause, 671 000 infections per year in the European Union alone ( Cassini *et al*., 2019) and a further 33, 000 deaths ( European Commission, 2018). In England, public health efforts to tackle AMR have led to declines in antibiotic use across healthcare settings, but rates of prescribing remain high compared to some other European countries ( ESPAUR, 2018). Most importantly, the number of drug-resistant infections continues to rise ( Cassini *et al*., 2019), highlighting the need for renewed efforts to improve the quality of antibiotic prescribing.

In the UK, the national action plan ( DHSC, 2019) outlines a range of approaches to reduce AMR. Antibiotic stewardship, defined as *“…an organisational or healthcare-system-wide approach to promoting and monitoring judicious use of antimicrobials to preserve their future effectiveness.*” ( NICE: 2015 p8) plays a critical role in this ( DoH, 2013; WHO, 2015). A wide range of interventions have been found to improve antibiotic stewardship when applied across diverse healthcare settings ( Davey *et al.*, 2015; Hulscher & Prins, 2017). Effect sizes vary markedly, however, with apparently similar interventions producing very different results ( Davey *et al*., 2017).

Behaviour change is key to combating AMR - in terms of appropriate antibiotic prescribing, infection prevention & control and use of diagnostics. Yet reviews to date show behavioural and social influences are often not given due consideration in design of stewardship programmes ( Charani *et al*., 2011). Rationales for intervention designs are often not given, and the range of behaviour change techniques used are limited ( Davey *et al*., 2017). There have thus been calls for the adoption of a multidisciplinary approach to designing stewardship interventions ( Hulscher & Prins, 2017).

Medical Research Council guidance advocates a systematic, theory-based approach to intervention development, piloting, and evaluation ( MRC, 2006), and a number of frameworks from the behavioural sciences have been developed to facilitate this. For instance, the Behaviour Change Wheel approach ( Michie *et al*., 2011; Michie *et al*., 2014) advocates three key steps in the intervention development process: step 1) defining the problem of interest in behavioural terms (i.e. who/what/where/when); step 2) conducting a behavioural diagnosis to identify the range of individual, socio-cultural and environmental factors influencing the behaviour; step 3) using findings from behavioural diagnosis as a basis for selecting interventions, strategies and components that are likely to target any barriers/enablers to behaviour change ( Michie *et al*., 2014).

Different disciplines offer evidence, theories, frameworks, and methods that hold the potential to facilitate each of these steps. For instance, to facilitate step 1: epidemiological analyses of antibiotic prescribing can inform more precise and evidence-based specification of the target behaviour by providing information that identifies and prioritises patient groups, types of prescribing, stage of the prescribing process (antibiotic initiation versus de-escalation) and healthcare professional roles involved in prescribing. To facilitate step 2: behavioural science offers integrated theories and frameworks, such as the Theoretical Domains Framework ( Michie *et al*., 2005) and the Capability, Opportunity, Motivation – Behaviour (COM-B) ( Michie *et al*., 2011) that summarise the individual, socio-cultural, and environmental influences on behaviour. These can be used to explore influences on the behaviour of interest, using a range of methodological approaches (qualitative interviews, surveys, evidence synthesis). This can be complemented by social science methods, such as ethnographic observations, that can help develop a deep contextual understanding of what drives current behaviours and what needs to be targeted to enable change. In particular, these methods allow us to think about why an intervention that works in a particular setting might fail in another or show remarkably different effects. To facilitate step 3: The Behaviour Change Technique (BCT) taxonomy v1 and Behaviour Change Wheel ( Michie *et al*., 2011; Michie *et al*., 2013) can identify and categorise component BCTs and intervention functions in existing interventions, and identify those associated with improved effectiveness. Lastly, these frameworks can be mapped against one another to suggest types of intervention strategies that are likely to be effective and relevant in addressing different types of barriers and enablers ( Cane *et al*., 2015; Michie *et al*., 2014).

**Figure 1.**
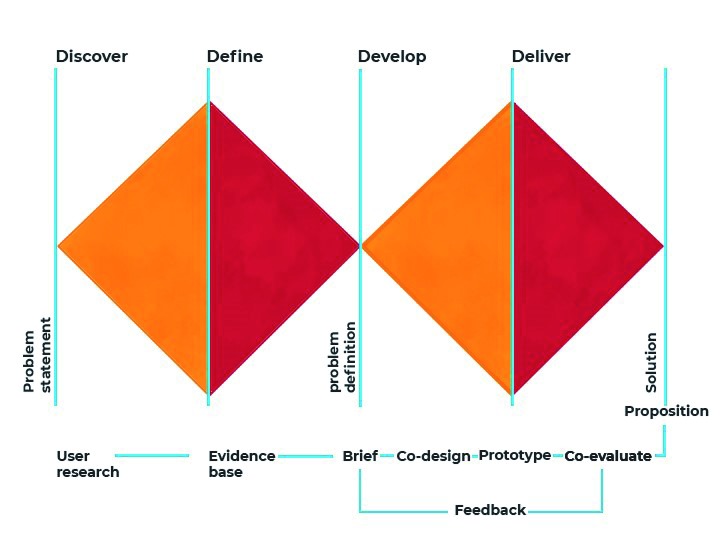
The Double diamond model.

Whilst the above frameworks can signpost types of interventions that are likely to be relevant, user-centred design methods can engage with potential users as experts, throughout the development period in order to ensure that stewardship interventions are relevant to stakeholders and designed in a way such that they can be successfully delivered and operationalised ( Chandler *et al*., 2016; Steen *et al*., 2011). This work can be guided by the Double Diamond design process model illustrated in Figure 1. This describes the design process in 4 phases, “Discover” (gathering insights) “Define” (frame the design question), “Develop” (testing solutions) and “Delivery” (production of the solution). This model has been widely applied to multidisciplinary design research and the generation and development of design solutions for user-centred problems ( Design Council, undated) – including health care challenges ( Chandler *et al*., 2016; Steen *et al*., 2011; West *et al*., 2017).

This protocol describes the structure and planned methods of the Preserving Antibiotics through Safe Stewardship (PASS) research programme, funded by the Economic and Social Research Council. The PASS interdisciplinary team involves researchers with expertise in design, psychology, behaviour change, social-science, and epidemiology. The project is a three-year programme of research structured around three sequential work-packages investigating antibiotic use in primary-care, secondary-care, care-homes, community pharmacy and amongst the general public.

The overarching aim of PASS is to identify patterns and drivers of antibiotic use across care settings, as a basis for developing theory-, and evidence-based antimicrobial stewardship interventions. Specific objectives and research questions for individual work-packages are outlined in the Methods section.

## Methods

### Study design

Mixed-methods study (see Table 1 below)

### Settings

Primary-care, secondary-care, care-homes, community, community pharmacy (see Table 1).

### Research question

Research questions for the three work-packages, data sources and settings are summarised in Table 1 below.

**Table 1.** Protocol for the Preserving Antibiotics through Safe Stewardship (PASS) research questions, presented according to work-package and applicability to care settings.

WORK-PACKAGE 1 (WP1) : IDENTIFYING PATTERNS OF ANTIBIOTIC PRESCRIBING					
Data sources: **Primary–care:** electronic health records (EHR), **secondary-care:** EHR, **Care-home:** Administrative data and pharmacy records, **Community (Bug Watch)**: online prospective survey, **Community pharmacy**: n/a					
Question	Primary- care	Secondary-care	Care-homes	Community	Pharmacy
What is the frequency of antibiotic prescribing?	✓	✓	✓	✓	n/a
Who gets antibiotics and why?	✓	✓	✓	✓	n/a
Is prescribing appropriate?	✓	✓	✓	n/a	n/a
What is the frequency of re-consultation or hospital admission	✓	n/a	n/a	n/a	n/a
What proportion of patients with symptoms consult their GP	n/a	n/a	n/a	✓	n/a
**WORK-PACKAGE 2 (WP2) – IDENTIFYING BARRIERS AND ENABLERS TO STEWARDSHIP IN CONTEXT**					
Data sources: **Primary–care, secondary-care, care-homes:** interviews, observations, design workshops, systematic review **Community** & **Community Pharmacy**: interviews, systematic review					
	Primary- care	Secondary-care	Care-homes	Community	Pharmacy
Interviews: What are the barriers to, and facilitators of, antibiotic prescribing?	✓	✓	✓	n/a	✓
Interviews: What drives patients to seek antibiotics for their symptoms?	n/a	n/a	n/a	✓	n/a
Observations: What are the contextual factors that underpin variations in prescribing?	✓	✓	✓	n/a	n/a
Observations and interviews: What are staff experiences of, attitudes towards and uptake of existing interventions to optimise prescribing?	✓	✓	✓	n/a	✓
Systematic Reviews: What interventions are effective in improving stewardship?	✓	✓	✓	✓	✓
Systematic Reviews: Which behaviour change techniques have been used in existing interventions, and which are associated with increased effectiveness?	✓	✓	✓	✓	✓
Design workshops: Which are the practical and feasible entry points for interventions?	✓	✓	✓	Data not collected	Data not collected
Design workshops: What are the constraints that impact the form of the intervention?	✓	✓	✓	Data not collected	Data not collected
**WORK-PACKAGE 3 (WP3)– CO-PRODUCTION OF INTERVENTION BUNDLES AND DISSEMINATION**					
Data sources: **All settings**: Triangulation of systematic review findings against findings from WP1 and WP2, stakeholder workshops.					
	Primary- care	Secondary-care	Care-homes	Community	Pharmacy
Triangulation: Do existing interventions target key drivers of antibiotic prescribing?	✓	✓	✓	✓	✓
Triangulation: What interventions are likely to be effective and relevant?	✓	✓	✓	✓	✓
Stakeholder workshops: What are stakeholder views on how bundles can best be delivered?	✓	✓	✓	✓	✓
Stakeholder workshops: Which interventions meet the APEASE criteria and should be prioritised?	✓	✓	✓	✓	✓

### Ethics approval and consent to participate

WP1: Use of CPRD was approved by the Independent Science Advisory Committee (ref ISAC-Nr: 17_048). Analysis of patterns of prescribing in secondary-care from a single Trust (using de-identified data) was registered as an audit and was not subject to ethical approval. The use of care-home data was approved by the UCL ethics committee (Ref: 11813/002) Ethical approval for the Bug Watch Survey was given by the UCL Ethics committee (ref: 11813/001)

WP2: Semi-structured interviews and observations and design-led workshops in care-homes, primary-care and secondary-care were ethically approved by the Westminster REC (ref: 235444). Semi-structured interviews with Bug Watch participants and community pharmacists were approved by UCL ethics committee (ref:13355/001).

Participants in the Bug Watch Survey and those taking part in semi-structured interviews will be required to provide informed consent before participating. All other parts of the study involve anonymised data and informed consent will not be required.

WORK-PACKAGE 1 (WP1). – IDENTIFYING PATTERNS OF ANTIBIOTIC PRESCRIBING

High quality information on prescribing patterns, the management of infection and clinical outcomes across healthcare settings in England is currently lacking. WP1 addresses this by evaluating antibiotic prescribing in primary-care, secondary-care and care homes through an epidemiological analysis of electronic health records, care-home administrative data and pharmacy records, and by developing an online survey of patients in the community, the “Bug Watch” survey ( Smith *et al*., 2019). These findings will provide insight into where problems lie and where intervening will be of greatest value. These analyses will also help define the target behaviours to be explored in interviews and observations in work-package 2, as well as sampling of participants/sites. Specific research questions for WP1 are presented in Table 1.

### Procedure

***Primary-care.*** A retrospective cohort study of patients registered with Clinical Practice Research Datalink (CPRD) practices, with a nested Cohort study of those presenting with common acute infections.

The CPRD is a primary-care dataset containing de-identified data from 11 million currently registered patients from a network of GP practices across the UK. All patients who were registered with a general practice between January 1st 2008 and December 31st 2015 are eligible for inclusion, provided their data met specified quality standards ( Herrett *et al*., 2015). A subset of the dataset is linked to information on hospital admissions through hospital episode statistics (HES) and census data through the Office of National Statistics. Using this dataset, we will extend an existing analysis ( Shallcross *et al*., 2017) and examine which patients (in terms of age, gender, comorbidities, socioeconomic variables, obesity, and smoking) receive the most antibiotics. This will identify which patients are most likely to receive antibiotics, for which infections and with what treatment regimes. These will be related to national guidance ( NICE, 2015) on primary-care antibiotic prescribing to assess appropriateness.

In the nested cohort study we will investigate adverse outcomes (risk of re-consultation, hospitalisation and death within 28 days) in patients who present to primary-care with specific clinical infection syndromes, comparing patients who were/were not treated with antibiotics.

Data analysis and statistical plan: Using statistical approaches such as propensity scores we will estimate the absolute risk and number-needed-to-treat (NNT) with antibiotics to prevent adverse outcomes.

***Care-homes.*** A descriptive analysis of routine data from a national chain of >300 care-homes, linked, at the level of individual residents, to antibiotic dispensing data for the same period will be carried out.

Prescribing data are supplied by a national chain of pharmacies; the sole provider of dispensing services to these care-homes and includes the type of drug, duration, dose and route of administration. Data from the care home are derived from a number of databases held by the care-home provider and includes information on incident reporting ( Datix), demographic characteristics of residents, and characteristics of the care-home (including staffing, resident numbers, resident types and location). Records will be extracted for individuals who were care-home residents between January 1st, 2016 and December 31st, 2017.

 Data analysis and statistical plan: We will identify the major infection categories leading to antibiotic use, the antibiotics used and the characteristics of residents receiving antibiotics. We will generate home-specific rankings of antibiotic use to divide homes into quintiles for sampling in WP2.

***Secondary-care.*** A descriptive analysis of data from electronic health records, derived from a large teaching hospital in central England will be carried out. This is a unique data-holding that includes high-resolution, anonymised data on symptoms, diagnoses, co-morbidities, prescriptions, test results, and vital signs, and information on long-term clinical outcome through linkage to death certifications and hospital episode statistics. Data will be extracted for every patient who was admitted to this hospital and prescribed at least one antibiotic between January 1st, 2011 and December 31st, 2016.

Data analysis and statistical plan: The analysis will include a description of the distribution of antibiotic prescribing according to clinical indication, patient group, clinical speciality, prescriber seniority and period. Clinical indications will be derived from symptoms, vital signs and biomarkers recorded during admission, and ICD-10 codes allocated at discharge from the hospital. We will investigate the appropriateness of high volume antibiotic prescribing in up to five clinical specialities; taking account of factors such as age and co-morbidities which might influence prescribing decisions. This work will inform selection of wards and teams for inclusion in WP2.

***Community/general population (Bug Watch Survey).*** A prospective community cohort study entitled Bug Watch will be carried out. We will invite 21745 adults who previously took part in the Health Survey for England, and agreed to be contacted for further research ( Smith *et al.*, 2019), along with children living in these households. All participants in the Health Survey for England in 2013, 2014 and 2015 who also agreed to be contacted re/ further research will be eligible for inclusion.

 Information on participants will be captured through an online survey which collects baseline information on participant characteristics including: demographics, presence of comorbidities, and knowledge of antibiotics. Once enrolled, participants will receive a weekly email over a six-month period inviting them to record possible symptoms of infection that they experienced in the prior week, such as coughs, fever, rash, diarrhoea. Those reporting symptoms will be asked to record whether they sought help from a healthcare provider, whether they requested and/or received antibiotics, and factors influencing their healthcare and antibiotic seeking behaviours based on the COM-B model ( Michie *et al*., 2011). Parents will be asked to complete surveys on behalf of children.

Study data will be collected and managed using REDCap electronic data capture tools hosted at University College London REDCap (Research Electronic Data Capture) is a secure, web-based software platform designed to support data capture for research studies ( Harris *et al*., 2009).

Data analysis and statistical plan: The proportions of people with common symptoms of infection who consult their GP and receive antibiotics will be estimated. We will investigate how these healthcare-seeking and treatment behaviours vary by age, gender, ethnicity, presence of other illnesses, social deprivation, symptom duration and severity.

WORK-PACKAGE 2 (WP2) – IDENTIFYING BARRIERS AND ENABLERS TO STEWARDSHIP IN CONTEXT

WP2 draws on theory, methods and principles from the behavioural and social sciences to investigate factors influencing antibiotic prescribing in context. Findings from WP1 will facilitate specification of the target behaviours to be investigated in WP2, by providing an evidence base for specifying who/what/where/when (i.e. identify target patient groups, the time frame, types of antibiotics, and prescribers). For each setting, we will conduct: 1) theory-based semi-structured interviews to identify barriers and enablers to appropriate antibiotic prescribing; 2) ethnographic observations to investigate socio-cultural and contextual factors shaping antibiotic use, 3) systematic reviews to specify the active ingredients (i.e. behaviour change techniques) of existing antimicrobial stewardship interventions and 4) designer led workshops to explore initial intervention generation. Specific research questions for WP2 are outlined in Table 1.

### Procedure

***Semi-structured interviews.*** Semi-structured interviews will be carried out with healthcare professionals in primary-care (4 sites in England), community pharmacy (up to 16 sites in England), secondary-care (4 wards/consultant teams in an English teaching hospital), and care-homes (4 homes in England) and with members of the public (recruited via the Bug Watch survey). Healthcare sites will include a mix of high and low prescribing sites. Care-homes and wards/consultant teams will be purposively sampled based on prescribing patterns data from WP1 and primary care sites, using data available from the Public Health England “Fingertips” platform. A purposive sample of Bug Watch participants, who agreed to be contacted, had symptoms of infection and did or did not seek antibiotics from their GP for experienced symptoms will be invited to participate. A convenience sample of pharmacists will be used.

We will conduct a minimum of 16 interviews per care setting (n= 8 from two high prescribing general practices/wards/care-homes, n = 8 from two low prescribing general practices/wards/care-homes, n=8 Bug Watch participants that sought antibiotics, n=8 Bug Watch participants that did not seek antibiotics, n=16 community pharmacists), 80 interviews in total. We will assess for thematic data saturation following the interviews, and conduct further interviews as needed until data saturation is achieved ( Francis *et al*., 2010).

The overall target behaviour to be explored in interviews conducted in healthcare settings is antibiotic use (including the sub-behaviours around prescribing, reviewing, switching, stopping antibiotics, advice giving, infection management). We will further specify this based on WP1 findings (e.g. prescribing of antibiotics for specific patient groups, types of antibiotics, times/days of week etc). The target behaviour to be explored in interviews conducted with Bug Watch participants is antibiotic and healthcare seeking for symptoms. Topic guides (see extended data ( Shallcross *et al.,* 2020) ) for all settings will be based on the Theoretical Domains Framework (TDF) ( Cane *et al*., 2012), which synthesises 33 behaviour change theories into 14 domains representing the range of individual, socio-cultural and environmental influences on behaviour. The topic guide will include at least 1 question per domain, and will be developed in collaboration with healthcare professionals, behavioural scientists and patient and public representatives. The topic guide will be piloted with at least two participants prior to data collection. Interviews lasting a maximum of one hour will be conducted either face to face or via telephone. Interviews will be audio-recorded, transcribed verbatim and fully anonymised so that no individual or organisation can be identified from the data. The researcher will record interviews using an encrypted recorder. This will then be transcribed by a University approved supplier, with a signed confidentiality and data management agreement in place. Once transcripts are completed and checked, recordings will be deleted.

Data analysis: Transcripts will be analysed using a combined deductive framework and inductive thematic analysis approach, in line with recently published guidance ( Atkins *et al*., 2017). Participant responses will be deductively coded into the TDF domain they are judged to best represent. Subsequently, similar responses within each domain will be grouped, and a theme label inductively generated. Theme labels will summarise the role that domain plays in either facilitating or hindering appropriate antibiotic use. Key domains will be identified using established criteria: frequency, expressed importance, and discord ( Atkins *et al*., 2017). Findings in high vs low prescribing participants will be compared, as well as according to professional roles (e.g. nurses vs physicians). Analysis will be conducted separately for each care setting first, and subsequently compared.

***Ethnographic observations.*** Ethnographic observations of practice will be undertaken in areas where semi-structured interviews have taken place (4 primary care practices, 4 nursing homes, and 4 secondary care consultant teams). We will develop a structured observation guide based on findings from WP1 and drawing on Bate’s framework of context in improvement to identify sensitising concepts ( Bate, 2014). A researcher will visit each site for up to five days and observe clinical practice, shadow staff, and collect local documents and guidelines.

The researcher will gather data on salient features of the local systems, social factors, and organisational context that may impact on local prescribing patterns. Data will be in the form of field notes from observations, audio recordings of the observer’s reflections and discussions with healthcare-workers, patients, and relatives. The researcher will record their impressions using an encrypted recorder. This will then be transcribed by a University approved supplier, with a signed confidentiality and data management agreement in place. Once transcripts are completed and checked, recordings will be deleted. Field notes and audio recordings will be transcribed verbatim and coded using NVivo software (version 12).

Data analysis: The analysis will draw on elements of grounded theory, in particular, the constant comparative approach ( Charmaz, 2014), and will aim to generate accounts of the influence of social and contextual factors on antibiotic use in different settings. We will use techniques including diagramming and theme summaries to synthesise our findings ( Charmaz, 2014). Analysis of interview and observational data will be supported through regular discussion between researchers to ensure that the findings can be brought together to generate a more comprehensive picture of influences on behaviour that will be taken into account in intervention design.

***Evidence synthesis.*** Systematic reviews of published antimicrobial stewardship interventions will be carried out for primary-care, secondary-care, care-homes, community pharmacy and general population. The reviews aim to establish the effectiveness of interventions targeting AMS and what content contributes to intervention effectiveness (see Table 1 for specific research questions). Review protocols will be registered on PROSPERO and will be conducted in accordance with PRISMA guidelines ( Moher *et al*., 2009). The systematic reviews will be conducted using EPPI-Reviewer version 4 ( EPPI-Centre, 2010): a comprehensive online software tool for research synthesis enabling management and analysis of literature review data. The software will be used to manage all stages of the process from bibliographic management, screening, coding, and synthesis.

We will first conduct a scoping search to identify existing reviews and/or registered protocols of reviews of antimicrobial stewardship interventions in primary-care, secondary-care, care-homes, and community pharmacy, plus interventions influencing antibiotic seeking and infection management in the general population. Where an existing review does not exist, we will conduct a systematic search of electronic data bases (Medline, PubMed, EMBASE, PsychInfo) and hand searches of reference lists, to identify published evaluations of interventions targeting antibiotic stewardship in the given care setting. Language will be restricted to English. No publication date restrictions will be imposed on the search algorithms. Where a recently published, comprehensive review exists, we will conduct a secondary analysis of interventions included in that review.

Titles, abstracts and full texts will be screened against pre-specified inclusion criteria (randomised trials, observational studies, systematic reviews) and exclusion criteria (qualitative studies, grey literature, case-studies). Of these, 25% will be double-screened by two reviewers at each step. Quality appraisal of all included studies will be undertaken using the Cochrane risk of bias tool (randomised studies) and the ROBINS-I tool (non-randomised studies).

The behavioural outcome of interest is antibiotic, - prescribing, -dispensing or -consumption. The outcome may be measured as the total amount prescribed, dispensed or consumed; or as the proportion of antibiotics appropriately prescribed, dispensed or consumed.

Data analysis and statistical plan: A pre-designed proforma will be used to extract data on study characteristics, behavioural outcomes and effect sizes. Data extraction will focus on the following: publication details, clinical/demographic characteristics, study design/methods, intervention types, outcomes, data-collection method and statistical analyses. Data on the behaviours being targeted will also be extracted: specified according to the TACTA framework Action, Actor, Context, Time-frame, Target ( Presseau *et al*., 2019). A full list of fields used for data extraction in the pro-forma are provided as extended data ( Shallcross *et al.,* 2020).

We will apply the Behaviour Change Wheel ( Michie *et al*., 2011) and the Behaviour Change Technique taxonomy v1 ( Michie *et al*., 2014) to identify and categorise the intervention functions and behaviour change techniques (BCTs) reported in the published descriptions of included interventions.

We will use descriptive statistics to summarise the use of intervention functions and BCTs across trials. A narrative synthesis will also be conducted. The impact of type and number of intervention functions and BCTs on effect size will be investigated

Where feasible, the data will be synthesised through meta-analysis using the random effects inverse variance method to establish pooled effect sizes for interventions and their components. The consistency of the results from each study will also be assessed by a forest plot and the degree of heterogeneity will be determined using the χ² test and the I² statistic.

We will also conduct meta-regressions in sub-group analyses to explore which specific intervention functions and BCTs are associated with improved effectiveness. Such findings can inform the selection of potential components to include in the design and/or refinement of future interventions to improve antibiotic stewardship. Such findings can inform the selection of potential components to include in the design and/or refinement of future interventions to improve antibiotic stewardship.

***Design-led workshops.*** Workshops will be carried out that will extend and complement the interviews and observations. We will work in 1–2 sites each in primary-care, secondary-care and care-homes, where interviews and observations have already been carried out. We will work with health care teams at selected sites, and invite participation from a variety of clinical, support and ancillary staff. Individual and group activities will engage healthcare staff in creative ways to understand their experience and expertise around antibiotic use. Workshops will employ a combination of inclusive-design research tools and techniques. Techniques will include imagining and drawing personal ideals; describing tasks or reflecting on strategies using visual worksheets; problem-solving or ideating impossible scenarios. A variety of tools will be used, such as playing cards to indicate feelings; voting or ranking tasks; simulation and substitution games for creative problem-solving. Inspiration for these have been drawn from a number of sources and disciplines, including creative thinking theorists such as De Bono, IDEO design group methods, human factors theory and practice, and inclusive design methodologies ( www.designingwithpeople.org, 2019).

WORK-PACKAGE 3 (WP3) – CO-PRODUCTION OF INTERVENTION BUNDLES AND DISSEMINATION

In WP3 PASS researchers will work with the public and healthcare practitioners to develop intervention bundles where there is scope to improve stewardship, employing structured-approaches commonly used in the behavioural sciences ( Michie *et al*., 2014) combined with principles of user-centred design ( Steen *et al*., 2011). An online platform to disseminate PASS outputs will also be developed.

### Procedure

Triangulation: We will host data triangulation workshops with research team members from each work-package in order to integrate and compare findings across work-packages. In the first instance, this will be done for each care setting individually. The aim of the workshops will be to identify commonalities and consistent themes and findings emerging from each work package, as well as areas of discord. Following published data triangulation procedures ( Farmer *et al*., 2006; Hopf *et al*., 2016; Tonkin-Crine *et al*., 2016), we will tabulate key findings from each work-package and record whether these findings are convergent with-, divergent from-, or absent from findings of other work-packages. We will look for consistent themes across data sources regarding patterns and influences on antibiotic prescribing in each care setting.

Analysis plan: Consistent themes across data sources represent potential challenges to be addressed by antimicrobial stewardship interventions. We will therefore design interventions to target these themes. First, we will follow the steps in the Behaviour Change Wheel approach to systematically identify types of intervention functions and BCTs that are theoretically congruent with, and likely to be relevant and effective in, addressing the key barriers and enablers identified in WP2. This will be done by consulting the mapping matrices ( Cane *et al*., 2015; Michie *et al*., 2014) which pair intervention functions from the Behaviour Change Wheel ( Michie *et al*., 2011) and BCTs from the taxonomy v1 ( Michie *et al*., 2014) with domains from the TDF ( Cane *et al*., 2012), on which the WP2 interviews are based. This will generate a long list of potential intervention components. We will prioritise amongst these by using evidence from the WP2 systematic reviews regarding which intervention functions and BCTs are associated with increased effectiveness in improving AMS behaviours. This will result in a final set of candidate intervention bundles.

Stakeholder workshops: Candidate intervention bundles will then be presented and discussed in a series of co-design workshops with key stakeholder representatives, such as healthcare professionals, policy makers, patient and public representatives, multidisciplinary researchers. Project staff will use research publications, personal contacts and knowledge of the field to identify relevant stakeholders, who will be invited via email. We will use the ‘people in research’ website to identify and invite patient/public representatives.

Stakeholders will be asked to discuss each bundle in turn using the APEASE criteria to help select between these potential interventions (i.e. Acceptability, Practicability, Effectiveness, Affordability, Side-Effects, Equity) ( Michie *et al*., 2014). To provide context and guide discussions, stakeholders will also be presented with summaries of the study findings. Nominal group techniques ( Harvey & Holmes, 2012), will be used to help reach consensus on which interventions are most likely to be appropriate, effective, and feasible to deliver in local context and ways of working.

Analysis plan: Following completion of the stakeholder workshops, the design team will develop the most promising intervention concepts through a series of design iterations. This will involve further co-design work with healthcare staff and stakeholders, whereby each concept will undergo a ‘design sprint’ moving from concept materialisation (prototyping, mock-ups), to user-testing and review. The design sprints will ensure that the intervention concept meets its goal, that micro and macro feasibility issues are identified and problem-solved, and that functionality and efficiency are secured as far as possible. PASS sprints will establish a meshing of inclusive design techniques (both research and design development) with LEAN ( Jauregui-Becker & Perry, 2015) or rapid iterative design processes ( Medlock *et al*., 2002). We will then develop intervention descriptions, protocols, and materials to take forward to formal pilot/feasibility testing and outcome evaluation.

### Dissemination of information

Anonymised summary data will be made available via the PASS website and will be searchable via the UK Data Service.

Summaries of available evidence on AMS across these settings, and the key findings from each WP in PASS will be made available as a resource for patients, the public, policymakers and healthcare professionals through an interactive website that will be developed in consultation with stakeholder panels. We will develop patient and public infographics supporting safe self-management of infection and shared decision-making. The website will also act as a practical guide for researchers, policy makers, and practitioners to help support selection and design of AMS intervention for different antibiotic use contexts and as a resource for researchers. Where appropriate the website will link to existing resources of information to avoid duplication of materials.

### Study status

Data collection for work-package one is complete and analysis is ongoing. Data collection and analysis for work-package 2 is ongoing. Expected date of project completion July 2020.

## Discussion

This programme of research will take an inter-disciplinary, theoretically and empirically-informed approach to developing AMS interventions across the UK healthcare economy. We will use quantitative and qualitative methods, design-led research, evidence synthesis, systematic approaches to behavioural intervention design, and evidence based participatory design principles, to design robust and effective theory-based interventions.

The knowledge generated by this programme of work will include extensive data that characterises infection management, infection outcomes, and antibiotic use, across community, primary-care, secondary-care, and nursing home settings. Quantitative data on patterns of antibiotic prescribing at an individual patient level are currently most sparse in nursing home-, and hospital-settings. Information on self-management of infections in the community and patterns of health-seeking behaviours is also lacking. We would expect these findings to be of particular interest to researchers, clinicians and policy-makers involved in the field of AMR, as well as to a wider-audience. Although our quantitative analyses of prescribing are of UK data we expect that scientists studying prescribing in different countries will be keen to use the data to make international comparisons. Measures of disease natural history and outcomes in treated and untreated patients will be broadly generalisable across high and middle-income settings.

Data on prescribing patterns will help us to more precisely specify the target behaviours for intervention, by revealing the most problematic areas of antibiotic over-use, help us define the problem in behavioural terms (for instance with whom, where and when to intervene) and to allow purposive sampling for WP2. This will enable effort to be focused on the points in care where there are the most gains to be made in intervening to improve antibiotic use. Generating comprehensive data on prescribing patterns and infection outcomes may also, in itself, open up new possibilities for intervention design that are dependent on such data.

The qualitative work will provide insights into professional and public behaviour including improved understanding of how people balance risks and benefits and the pressures that make it difficult to adhere to national and local guidance. We will structure our findings and our review evidence using increasingly applied theoretical frameworks from the behavioural and social sciences ( Michie *et al*., 2014). This approach will enable researchers to draw comparisons with our research more readily. We anticipate these findings will also be of relevance to researchers studying health care seeking and treatment behaviours in non-infection-related areas.

Our systematic approach to intervention development will allow other researchers who wish to test the effectiveness of stewardship approaches to select candidate intervention bundles. Our website will support this process by highlighting what behaviours need changing in different contexts, highlighting important drivers of these behaviours, showing what has been tried previously and suggesting combinations of interventions that are likely to work.

The planned work is highly inter-disciplinary, involving statisticians, epidemiologists, psychologists, ethnographers, designers and clinicians. This approach to the understanding of antibiotic prescribing from quantitative and behavioural science perspectives is a major strength of the study, particularly when it comes to developing innovative solutions to the problem of antibiotic stewardship. However, it also presents significant challenges. In particular, we will need to take into account the different methods and goals of a multi-disciplinary team. As such the work is likely to demonstrate a degree of complexity that will require a collegiate approach to management and leadership.

## Conclusion

This protocol describes a complex programme of inter-disciplinary research which aims to co-design theory- and evidence-based antibiotic stewardship interventions targeted to specific healthcare settings. Our outputs will be of value to a wide range of stakeholders including: patients, the public, researchers from a range of disciplines (including behavioural science, epidemiology, health informatics, health intervention design, health service researchers, trialists and mathematical modellers) within the UK and beyond, as well as healthcare workers and policy makers. Beyond this study, our aim is to pilot and trial the stewardship interventions that have been agreed to be most promising by our stakeholder panel, working towards implementation of effective AMS strategies in routine clinical practice.

## Data availability

### Underlying data

No data are associated with this article

### Extended data

Figshare: An interdisciplinary mixed-methods approach to developing antimicrobial stewardship interventions: Protocol for the Preserving Antibiotics through Safe Stewardship (PASS) Research Programme: Supplementary materials. https://doi.org/10.5522/04/11548497.v1 ( Shallcross *et al.,* 2020)

This project contains the following extended data:

- PASS_protocol_supplementary_materials.docx (Document containing interview schedules for primary-care, secondary-care and care-homes and the systematic review proforma for data extraction)

Data are available under the terms of the Creative Commons Zero "No rights reserved" data waiver (CC0 1.0 Public domain dedication).

## References

[ref-2] Atkins L Francis j Islam R: A guide to using the Theoretical Domains Framework of behaviour change to investigate implementation problems. *Implement Sci.* 2017;12(1):77. 10.1186/s13012-017-0605-9 28637486 PMC5480145

[ref-3] Bate P: Context is everything.In *Perspectives on context a selection of essays considering the role of context in successful quality improvement* London: Health Foundation.2014 Reference Source

[ref-5] Cane J O'Connor D Michie S: Validation of the theoretical domains framework for use in behaviour change and implementation research. *Implement Sci.* 2012;7(1):37. 10.1186/1748-5908-7-37 22530986 PMC3483008

[ref-6] Cane J Richardson M Johnston M: From lists of behaviour change techniques (BCTs) to structured hierarchies: comparison of two methods of developing a hierarchy of BCTs. *Br J Health Psychol.* 2015;20(1):130–50. 10.1111/bjhp.12102 24815766

[ref-7] Cassini A Högberg LD Plachouras D: Attributable deaths and disability-adjusted life-years caused by infections with antibiotic-resistant bacteria in the EU and the European Economic Area in 2015: a population-level modelling analysis. *Lancet Infect Dis.* 2019;19(1):56–66. 10.1016/S1473-3099(18)30605-4 30409683 PMC6300481

[ref-9] Chandler CIR Burchett H Boyle L: Examining Intervention Design: Lessons from the Development of Eight Related Malaria Health Care Intervention Studies. *Health Syst Reform.* 2016;2(4):373–388. 10.1080/23288604.2016.1179086 31514719 PMC6176770

[ref-8] Charani E Edwards R Sevdalis N: Behavior change strategies to influence antimicrobial prescribing in acute care: a systematic review. *Clin Infect Dis.* 2011;53(7):651–62. 10.1093/cid/cir445 21890770

[ref-10] Charmaz K: Constructing Grounded Theory, 2 ^nd^ Edn.London: Sage.2014 Reference Source

[ref-12] Davey P Marwick CA Scott CL: Interventions to improve antibiotic prescribing practices for hospital inpatients. *Cochrane Database Syst Rev.* 2017;2:CD003543. 10.1002/14651858.CD003543.pub4 28178770 PMC6464541

[ref-11] Davey P Peden C Charani E: Time for action-Improving the design and reporting of behaviour change interventions for antimicrobial stewardship in hospitals: Early findings from a systematic review. *Int J Antimicrob Agents.* 2015;45(3):203–12. 10.1016/j.ijantimicag.2014.11.014 25630430

[ref-15] DHSC: Tackling antimicrobial resistance 2019-2024 The UK’s five-year national action plan.London: Department of Health and Social Care.2019 Reference Source

[ref-14] DoH 2013: UK Five Year Antimicrobial Resistance Strategy 2013 to 2018.London Department of Health.2013 Reference Source

[ref-16] Eppi-Centre: Undated Eppi-Reviewer.4 ed. Institute of Education University College London: Eppi-Centre-Social Science Research Unit UCL.2010 Reference Source

[ref-17] ESPAUR: English Surveillance Programme for Antimicrobial Utilisation and Resistance (ESPAUR): Report 2018.London: PHE.2018 Reference Source

[ref-18] European Commission: AMR: a major European and Global challenge.2018 Reference Source

[ref-19] Farmer T Robinson K Elliott SJ: Developing and implementing a triangulation protocol for qualitative health research. *Qual Health Res.* 2006;16(3):377–94. 10.1177/1049732305285708 16449687

[ref-20] Francis JJ Johnston M Robertson C: What is an adequate sample size? Operationalising data saturation for theory-based interview studies. *Psychol Health.* 2010;25(10):1229–45. 10.1080/08870440903194015 20204937

[ref-23] Harris PA Taylor R Thielke R: Research electronic data capture (REDCap)--a metadata-driven methodology and workflow process for providing translational research informatics support. *J Biomed Inform.* 2009;42(2):377–381. 10.1016/j.jbi.2008.08.010 18929686 PMC2700030

[ref-24] Harvey N Holmes CA: Nominal group technique: an effective method for obtaining group consensus. *Int J Nurs Pract.* 2012;18(2):188–94. 10.1111/j.1440-172X.2012.02017.x 22435983

[ref-25] Herrett E Gallagher AM Bhaskaran K: Data Resource Profile: Clinical Practice Research Datalink (CPRD). *Int J Epidemiol.* 2015;44(3):827–836. 10.1093/ije/dyv098 26050254 PMC4521131

[ref-26] Hopf YM Francis J Helms PJ: Core requirements for successful data linkage: an example of a triangulation method. *BMJ Open.* 2016;6(10):e011879. 10.1136/bmjopen-2016-011879 27797999 PMC5093676

[ref-27] Hulscher MEJL Prins JM: Antibiotic stewardship: does it work in hospital practice? A review of the evidence base. *Clin Microbiol Infect.* 2017;23(11):799–805. 10.1016/j.cmi.2017.07.017 28750920

[ref-4] Jauregui-Becker JM Perry N: Lean Design.In:, Laperrière L Reinhart G. (eds). CIRP Encyclopedia of Production Engineering. Springer, Berlin, Heidelberg.2015 10.1007/978-3-642-35950-7_16783-1

[ref-28] Medlock MC Wixon D Terrano M: Using the RITE method to improve products: A definition and a case study. Presented at the Usability Professionals Association 2002, Orlando Florida.2002 Reference Source

[ref-32] Michie S Atkins L West R: The behaviour change wheel. A guide to designing interventions. 1st ed. Great Britain: Silverback Publishing.2014 Reference Source

[ref-29] Michie S Johnston M Abraham C: Making psychological theory useful for implementing evidence based practice: a consensus approach. *Qual Saf Health Care.* 2005;14(1):26–33. 10.1136/qshc.2004.011155 15692000 PMC1743963

[ref-31] Michie S Richardson M Johnston M: The behavior change technique taxonomy (v1) of 93 hierarchically clustered techniques: building an international consensus for the reporting of behavior change interventions. *Ann Behav Med.* 2013;46(1):81–95. 10.1007/s12160-013-9486-6 23512568

[ref-30] Michie S Van Stralen MM West R: The behaviour change wheel: a new method for characterising and designing behaviour change interventions. *Implement Sci.* 2011;6(1):42. 10.1186/1748-5908-6-42 21513547 PMC3096582

[ref-33] Moher D Liberati A Tetzlaff J: Preferred reporting items for systematic reviews and meta-analyses: the PRISMA statement. *PLoS Med.* 2009;6(7):e1000097. 10.1371/journal.pmed.1000097 19621072 PMC2707599

[ref-34] MRC: Developing and evaluating complex interventions. MRC.2006 Reference Source

[ref-35] National Institute for Health and Care Excellence: Antimicrobial stewardship: systems and processes for effective antimicrobial medicine use NICE. NICE Guideline [NG15].2015 Reference Source

[ref-36] Presseau J McCleary N Lorencatto F: Action, actor, context, target, time (AACTT): a framework for specifying behaviour. *Implement Sci.* 2019;14(1):102. 10.1186/s13012-019-0951-x 31806037 PMC6896730

[ref-37] Shallcross L Beckley N Rait C: Antibiotic prescribing frequency amongst patients in primary care: a cohort study using electronic health records. *J Antimicrob Chemother.* 2017;72(6):1818–1824. 10.1093/jac/dkx048 28333200 PMC5437523

[ref-38] Shallcross L Lorencatto F Fuller C: An interdisciplinary mixed-methods approach to developing antimicrobial stewardship interventions: Protocol for the Preserving Antibiotics through Safe Stewardship (PASS) Research Programme: Supplementary materials. *figshare.*Workflow.2020 10.5522/04/11548497.v1 PMC7014923 32090173

[ref-39] Smith CM Conolly A Fuller C: Symptom reporting, healthcare-seeking behaviour and antibiotic use for common infections: protocol for Bug Watch, a prospective community cohort study. *BMJ Open.* 2019;9(5):e028676. 10.1136/bmjopen-2018-028676 31123004 PMC6537990

[ref-40] Steen M Manschot M De Koning N: Benefits of Co-design in Service Design Projects. *Int J Des.* 2011;5:2 Reference Source

[ref-13] The Design Council, Undated: What is the framework for innovation? Design Councils evolved Double Diamond. Reference Source

[ref-41] Tonkin-Crine S Anthierens S Hood K: Discrepancies between qualitative and quantitative evaluation of randomised controlled trial results: achieving clarity through mixed methods triangulation. *Implement Sci.* 2016;11:66. 10.1186/s13012-016-0436-0 27175799 PMC4866290

[ref-42] West J Meldaikyte G Raby E: Developing the Double Diamond Process for Implementation- insights from a decade of Inclusive Design projects. In Seemann K and Barron D. (Eds.). (2017). Design4Health, Melbourne. *Proceedings of the Fourth International Conference on Design4Health. Melbourne Cricket Ground, 4 – 7 Dec* 2017;308–310. Reference Source

[ref-43] WHO: Global Action Plan on Antimicrobial Resistance. Geneva: WHO.2015 Reference Source

