# Supplementary material for: An interdisciplinary mixed-methods approach to developing antimicrobial stewardship interventions: Protocol for the Preserving Antibiotics through Safe Stewardship (PASS) Research Programme
Source: Wellcome Open Res. 2020 Jan 14;5:8. [Version 1] doi: 10.12688/wellcomeopenres.15554.1 (PMC7014923; doi:10.12688/wellcomeopenres.15554.1)
Supplement: Peer review history

# Peer review: Reviewer response for version 1

**DOI:** 10.21956/wellcomeopenres.17033.r37697

**Published:** 2020-02-10

**Authors:** Judith Dyson

**Affiliations:** 1 City University London, London, UK

The authors clearly express the importance of optimal antimicrobial care in care homes, primary and secondary care. This broad study seeks to survey antibiotic seeking behaviours of the general public, through observations and interviews,  identify barriers and enablers to appropriate prescribing, conduct systematic reviews of interventions to support antimicrobial stewardship programmes and through this identify intervention components, co-design intervention(s) to support antimicrobial stewardship. The theoretical underpinning of this piece of work is the TDF.  

 This is a clear and well written protocol. However, there is a degree of complexity that is difficult to follow without several reads of the paper.  I would recommend a flow diagram to guide the reader through the study. 

 I wish the study team the very best of luck with this interesting and valuable piece of work.

I confirm that I have read this submission and believe that I have an appropriate level of expertise to confirm that it is of an acceptable scientific standard.

# Peer review: Reviewer response for version 1

**DOI:** 10.21956/wellcomeopenres.17033.r37581

**Published:** 2020-01-31

**Authors:** Paul Flowers

**Affiliations:** 1 MRC/CSO Social and Public Health Sciences Unit, University of Glasgow, Glasgow, UK

The **rationale** for, and **objectives** of, the programme are very clearly described. As an ambitious, interdisciplinary, multi-method and multi-discipline project the objectives are wide ranging and, as the authors note, they may prove challenging.

 The programme design is **highly appropriate** for the research questions. It involves the iterative development of intervention ideas through harmonising the relative strengths of a number of disciplinary perspectives and analytic lenses. These will be combined to produce a number of useful, evidence-based, theoretically-informed and co-designed outputs. These are very well placed to be able to reduce the drivers of antimicrobial resistance.

 In the main there are sufficient details of the methods provided. However, I believe there is some room for **further clarity** at a number of points across the manuscript.

 Personally, I did not find Figure 1 (Double diamond model) helpful in understanding the programme and the development of knowledge therein. It doesn't show which work packages were being reflected within each stage (i.e., discover, design, develop). To me the current figure seems to be independent of much of the behavioural work detailed within the protocol. Could this be revisited and attuned to the specific programme of research? Should it attempt to depict the flow of knowledge across the programme and its work packages and how such knowledge is realised across the various settings?

 One key area where there is a lack of clarity is the approach to synthesising or integrating the findings of the diverse methods within **work package 3**. It may be that specific and detailed future intervention development protocols are planned but I think the current protocol lacks some potentially important details for the reader to understand and anticipate some of the potential challenges within the project. From my understanding of the programme, work package 3 must marry the granular outputs from previous work packages with the more practical insights and expertise of the stakeholders. How are the team considering how to manage this? Will some of what has been gained from the granularity of approach be lost? On a more practical level, how many participants will there be within this work package, which sampling approach will be taken to recruit them, how will there particular expertise be weighted? How will the specific language of the behavioural science (eg. BCTs and TDF domains) be translated and made meaningful for the workshop attendees?

 I also felt there was an unexplained shift in the pitch of intervention content as the programme develops. The protocol moves from the detailed understanding of what interventions could be made from (their evidence-based and theoretically informed putative active ingredients, such as intervention functions, TDF domains or BCTs), to the more general term ‘intervention bundles’. Sorry if I have missed the explanation, and a rationale for the shift in the loci of change, or how it is operationalised across all the relevant health care settings but I found this shift to be a bit confusing. The team appear pragmatic in relation to behavioural specificity across the programme and I wonder if a pragmatic approach here is being planned too? More detail would help.

 Equally, some of the methods proposed here (i.e., nominal group techniques) may focus too inwardly on understanding intervention components able to moderate prescribing behaviour. Perhaps the adoption of this approach alone neglects the incorporation of stakeholder insights into future implementation and wider health care systems-level phenomena. On a more basic level, it would be helpful to have a little more explanatory detail for approaches such as ‘LEAN’ or ‘design Sprints’.

 Again, as a minor point, I wondered about the Bug Watch Survey and whether information about pets and antimicrobials was being recorded.

I confirm that I have read this submission and believe that I have an appropriate level of expertise to confirm that it is of an acceptable scientific standard.
